# Prevention of Cellular Suicide by Cytomegaloviruses

**DOI:** 10.3390/v4101928

**Published:** 2012-10-02

**Authors:** Patricia M. Fliss, Wolfram Brune

**Affiliations:** Heinrich Pette Institute, Leibniz Institute for Experimental Virology, Martinistr. 52, 20251 Hamburg, Germany; Email: patricia.fliss@hpi.uni-hamburg.de

**Keywords:** HCMV, UL36, UL37x1, UL38, m41.1, M45, m38.5, M36, β2.7, necroptosis

## Abstract

As intracellular parasites, viruses rely on many host cell functions to ensure their replication. The early induction of programmed cell death (PCD) in infected cells constitutes an effective antiviral host mechanism to restrict viral spread within an organism. As a countermeasure, viruses have evolved numerous strategies to interfere with the induction or execution of PCD. Slowly replicating viruses such as the cytomegaloviruses (CMVs) are particularly dependent on sustained cell viability. To preserve viability, the CMVs encode several viral cell death inhibitors that target different key regulators of the extrinsic and intrinsic apoptosis pathways. The best-characterized CMV-encoded inhibitors are the viral inhibitor of caspase-8-induced apoptosis (vICA), viral mitochondrial inhibitor of apoptosis (vMIA), and viral inhibitor of Bak oligomerization (vIBO). Moreover, a viral inhibitor of RIP-mediated signaling (vIRS) that blocks programmed necrosis has been identified in the genome of murine CMV (MCMV), indicating that this cell death mode is a particularly important part of the antiviral host response. This review provides an overview of the known cell death suppressors encoded by CMVs and their mechanisms of action.

## 1. Introduction

Viruses are obligate intracellular parasites whose successful propagation depends on host cell functions. For every single step of the viral life cycle, from viral entry, to replication, assembly, and release of new virions, various host cell factors are needed. Moreover, the synthesis of viral components relies on the host cell. Therefore, successful viral replication and spread is only guaranteed if the host cell stays vital for a sufficient time. One strategy organisms have evolved to restrict the spread of viruses, is the induction of programmed cell death (PCD). By sacrificing infected cells, the host efficiently blocks virus replication. Viruses, on the other hand, express cell death inhibitors, which they use to sustain viability and life span of the host cell. 

Apoptosis is the best-studied and best-known form of PCD. Removing cells by apoptosis is important for different physiological processes such as embryonic development, tissue homeostasis, and immune response [1]. Characteristic morphological features of apoptosis include chromatin condensation, plasma membrane blebbing, reduction of cellular volume, and the formation of apoptotic bodies [2], which are membrane-enclosed vesicles that contain cellular constituents and are rapidly phagocytosed *in vivo*. Thus, intracellular material is not released during the apoptotic process and inflammatory responses are prevented. On the biochemical level apoptosis is associated with exposure of phosphatidylserine on the outer layer of the plasma membrane, cleavage of nuclear DNA, a decrease in mitochondrial transmembrane potential, and the cleavage of caspases [1]. Apoptosis is a highly regulated and ordered process that can be generally induced in two distinct ways: either by external signals (extrinsic pathway of apoptosis) or by intracellular stress conditions (intrinsic pathway) [3]. The intrinsic pathway can be triggered, for instance, by DNA damage, accumulation of unfolded proteins in the endoplasmic reticulum (ER) (ER stress), or microbial infections. These stress signals cause the activation of pro-apoptotic proteins of the Bcl-2 protein family in the mitochondria, which leads to mitochondrial outer membrane permeabilization (MOMP) and subsequently to the release of pro-apoptotic factors, such as cytochrome *c* (cyt *c*), Smac/DIABLO, or Htr2A/Omi from the mitochondria into the cytosol. Subsequently, these factors cause the activation of caspases, a family of aspartate-specific cysteine proteases that have key functions during apoptosis. Initiator caspases (e.g., caspase-8, -9, and -10) transduce death signals by activating effector caspases (e.g., caspase-3 and -7), which subsequently cleave various cellular substrates ultimately leading to the characteristic morphological and biochemical features of apoptosis [4]. The extrinsic apoptosis pathway is activated following stimulation of death receptors and leads to the formation of the death-inducing signaling complex (DISC), a protein complex responsible for activation of initiator caspase-8 [3]. In some cell types (so-called type I cells) caspase-8 directly causes cleavage of effector caspase-3 without the contribution of mitochondria. In other cells (type II cells) the DISC mediates apoptosis by activating the mitochondria-dependent pathway [5]. 

Necrosis has long been considered as an unregulated cell death form with morphological characteristics differing from those of apoptosis. For instance, necrotic cells show increased cell volume and swollen organelles. They lose membrane integrity leading to cell lysis and the release of cellular material into the extracellular environment, which may provoke an inflammatory response [6]. Only in recent years it has been revealed that necrosis, just as apoptosis, can be executed as a programmed process that is regulated by specific intracellular signaling pathways [7,8]. This form of necrosis has been classified as a type of PCD and was termed programmed necrosis, regulated necrosis, or necroptosis [3,9]. Programmed necrosis may function as a back-up cell death pathway, activated under certain conditions and in certain cells [10,11,12]. Triggers for programmed necrosis include cytokines, physico-chemical stress, or pathogens. [13]. The molecular regulation of programmed necrosis is poorly understood, but it has recently been revealed that kinases of the receptor-interacting protein (RIP) family are essential for intracellular transmission of necrotic death signals [8,14].

Apart from apoptosis and programmed necrosis, autophagic cell death has been proposed as a separate form of PCD. It is characterized by a massive accumulation of vacuoles (autophagosomes) in the cytoplasm of dying cells. However, it is still under debate whether these cells actually die by autophagy or whether cell death is only accompanied by this process [3,15]. Therefore, we will not further discuss autophagic cell death in this review. 

Human cytomegalovirus (HCMV) is a member of the *Herpesviridae* family. It is widely distributed in the adult population with infection rates ranging from 50% to nearly 100% [16]. HCMV can cause severe diseases in immunocompromised persons and immunologically immature individuals and persists life-long in the human body [17]. Since the virus is highly species specific, HCMV cannot be studied in small animal models. Instead, related viruses of mice and rats (MCMV or RCMV, respectively) are used as surrogates to study virus replication and pathogenesis *in vivo* [18,19,20]. CMVs are characterized by a protracted replication cycle lasting 48 to 72 hours in case of HCMV and about 24 hours in case of MCMV. This protracted replication cycle forces the virus to keep the host cell alive for a sufficient period of time. To achieve this, the CMVs have incorporated several cell death inhibitors into their genomes [21]. In this review we give an overview of the known CMV cell death inhibitors and the mechanisms of cell death inhibition.

## 2. Inhibition of Apoptosis at the Level of Mitochondria

**Figure 1:**
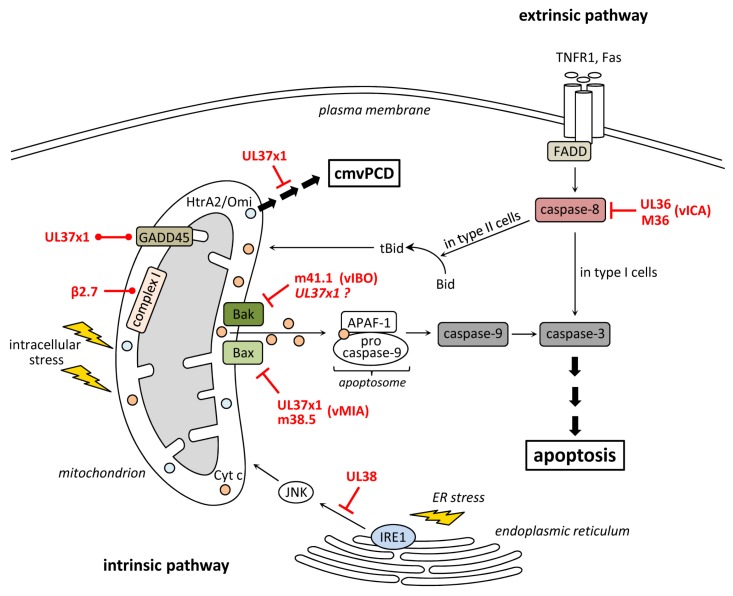
Inhibition of apoptosis and cmvPCD by human and murine cytomegalovirus (CMV). Ligation of death receptors (e.g., TNFR1 or Fas) leads to the activation of caspase-8, which directly activates caspase-3 (in type I cells) or cleaves Bid to tBid (in type II cells) inducing a mitochondrial signal amplification through the release of cyt *c*, which induces apoptosome formation followed by caspase-9 and -3 activation. The HCMV protein UL36 and its MCMV homolog M36 bind caspase-8 and inhibit its activation thereby suppressing death receptor-induced apoptosis. The HCMV protein UL37x1 and m38.5 of MCMV bind and inhibit the pro-apoptotic protein Bax. The anti-apoptotic function of UL37x1 is enhanced by GADD45 which is also bound by UL37x1. UL37x1 also delays the onset of an HtrA2/Omi-mediated cell fragmentation process (cmvPCD), which terminates CMV infection after an extended period of virus production. The HCMV β2.7 RNA interacts with complex I of the respiratory chain and stabilizes its function thereby maintaining ATP production and preserving metabolic activity during stress conditions. The HCMV UL38 protein suppresses ER stress-induced apoptosis.

Mitochondria have key functions during the intrinsic apoptosis pathway. They represent a platform at which pro- and anti-apoptotic signals converge. When lethal signals prevail MOMP is induced, which goes along with a loss of mitochondrial transmembrane potential, cessation of mitochondrial ATP synthesis, accumulation of reactive oxygen species (ROS), and release of pro-apoptotic factors from the mitochondrial intermembrane space into the cytosol [22]. Among them cyt *c* is the major “killing factor” since it induces the formation of the apoptosome, a multimeric protein complex consisting of procaspase-9, APAF-1 (apoptotic protease activating factor-1) and cyt *c* [23,24]. The apoptosome is responsible for proteolytic maturation of procaspase-9, which subsequently activates effector caspases inducing the execution phase of apoptosis (Figure 1). 

The induction of MOMP is tightly controlled by the Bcl-2 protein family, which consists of pro- and anti-apoptotic members that interact with each other. According to the number of Bcl-2 homology (BH) domains, family members can be divided into multidomain proteins containing 2 to 4 BH domains, and BH3-only proteins, which lack all but the BH3 domain [25]. The BH3-only proteins are pro-apoptotic and act as sensors or mediators for cellular stress [26]. According to the current model, activated BH3-only proteins neutralize anti-apoptotic multidomain proteins such as Bcl-2 and Bcl-x_L_ displacing them from the pro-apoptotic multidomain effector proteins Bax and Bak [27,28]. In addition BH3-only proteins may also directly bind and activate Bax and Bak [29,30,31]. Subsequently, Bax and Bak change their conformation, oligomerize in the mitochondrial outer membrane, and induce membrane permeabilization und subsequent apoptosis [32,33,34,35,36,37]. 

HCMV blocks apoptosis at the mitochondrial checkpoint by vMIA (viral mitochondrial inhibitor of apoptosis), a viral protein encoded by open reading frame (ORF) UL37 exon 1 (UL37x1). vMIA is a broadly acting cell death inhibitor that was shown to block apoptosis triggered by extrinsic as well as intrinsic stimuli [38,39,40,41,42,43]. At the biochemical level vMIA blocks mitochondrial release of cyt *c* as well as downstream processes such as cleavage of procaspase-9 and PARP (poly[ADP-ribose] polymerase). Activation of caspase-8 and Bid cleavage, however, is not blocked. It was shown that vMIA predominantly localizes to mitochondria, where it is inserted into the outer membrane [41]. Together these data indicated that vMIA acts at the level of mitochondria, reminiscent of Bcl-2. Despite the functional similarities to Bcl-2, vMIA shows no obvious sequence homology to this protein and lacks sequences similar to the BH domains [41]. More recently, however, an *in silico* structural prediction suggested that the overall fold of vMIA might be similar to Bcl-x_L_ [44]. 

The molecular mechanism of cell death inhibition by vMIA has been studied quite extensively. It was shown that vMIA interacts with Bax and recruits it to mitochondria [40], induces its oligomerization and tight association with mitochondria, possibly by its insertion into the outer membrane [45]. Although this status of Bax is usually associated with MOMP, MOMP is blocked in vMIA-expressing cells suggesting that vMIA sequesters Bax in an inactive state. vMIA’s capability to block cell death correlates with its ability to bind Bax and recruit it to mitochondria [45]. Two domains of vMIA are required for its anti-apoptotic function: a region spanning the N-terminal aminoacids 5 to 34, which largely overlaps with a mitochondria localization domain, and a region spanning the carboxy-terminal aminoacids 118 to 147, which is required for interaction with Bax and was designated the anti-apoptotic domain (AAD). Both domains together are necessary and sufficient for blocking apoptosis [41,46]. The AAD of vMIA also mediates interaction with the DNA damage response protein GADD45α (growth arrest and DNA damage 45 alpha) [47]. GADD45α as well as GADD45β and GADD45γ enhance the anti-apoptotic function of vMIA, although GADD proteins alone did not influence cell death under the conditions used. GADD45α also enhances the anti‑apoptotic function of Bcl-x_L_, which, interestingly, coprecipitated with vMIA as well. Experimental evidence suggested that GADD45α inhibits proteasome-dependent degradation of vMIA, thereby increasing the amount of the protein inside the cell [47]. Thus, in addition to vMIA’s interaction with Bax, complex formation with GADD45α and possibly also with Bcl-x_L_ seem to contribute to the anti-apoptotic activity of this cell death inhibitor. 

Besides blocking caspase-dependent apoptosis, vMIA controls a caspase-independent cell fragmentation process that occurs late during infection with the HCMV strain Towne and terminates CMV infection after a prolonged period of virus production [48]. This process was termed CMV‑infected cell-specific programmed cell death (cmvPCD) and is induced by HtrA2/Omi, a cellular serine protease that resides in mitochondria. During infection, vMIA appears to delay the onset of cmvPCD for several days. However, the underlying molecular mechanism remains largely unknown [48]. 

Other primate CMVs, such as those of rhesus macaques, chimpanzees, or African green monkeys, encode sequence homologs to vMIA. In case of rhesus CMV the anti-apoptotic function of its homolog has already been confirmed [49]. In contrast to primate CMVs, rodent and other CMVs do not encode obvious sequence homologs to UL37x1. However, MCMV expresses a protein with functional similarities to vMIA. This protein is encoded by ORF m38.5 that is located at an analogous position to ORF UL37x1 within the viral genome [43]. As shown for vMIA, the m38.5 protein interacts with Bax and recruits it to mitochondria [50], leading to its tight association with mitochondria during infection [51]. The m38.5 protein inhibits Bax- but not Bak-mediated apoptosis and, in accordance with this, no interaction with Bak was detected, classifying m38.5 as a Bax-specific inhibitor [50,52]. Different studies indicated that Bax and Bak play redundant roles during apoptosis meaning that the activity of either protein is sufficient to induce cell death [53,54]. Thus, in order to block mitochondrial mediated apoptosis completely, CMVs should be able to inhibit the activation of both, Bax and Bak. Indeed, it was shown that MCMV-infected cells are protected from Bak-mediated apoptosis [52,55]. This observation led to the search for and eventually the identification of an MCMV-encoded Bak inhibitor which is encoded by ORF m41.1 [56]. The mitochondrion-localized m41.1 protein interacts with Bak and inhibits its oligomerization, thereby blocking Bak-dependent cyt *c* release and apoptosis. Therefore, the m41.1 protein was named viral inhibitor of Bak oligomerization (vIBO) [56]. Thus, MCMV inhibits the mitochondrial pathway of apoptosis by two separate proteins. This principle of cell death inhibition seems to be conserved among rodent CMVs as sequence homologs of m38.5 and m41.1 were also found in two CMVs of rats [52,56]. Whether HCMV also encodes a Bak inhibitor remains unknown. One study showed that HCMV vMIA inhibits Bax- but not Bak-mediated apoptosis in murine cells [40], suggesting that HCMV might encode a separate Bak‑inhibitor. However, a sequence homolog to m41.1 is not present in the HCMV genome. Other reports questioned vMIAs Bax specificity by showing that vMIA also interacts with Bak and inhibits apoptosis induced by Bak overexpression [50,57]. Thus, vMIA might be capable of blocking both Bax- and Bak-mediated apoptosis. Others have argued that Bax might dominate over Bak in cell types that support HCMV infection. In such a scenario, Bax-inhibition by vMIA could be sufficient for blocking cell death at the mitochondrial checkpoint [40]. In sum, further studies are needed to answer the question whether or not HCMV is in need of a Bak-specific inhibitor and, if so, which viral protein fulfills this function.

It can be assumed that the anti-apoptotic activity of viral genes is required for efficient viral replication since premature cell death of infected cells should prevent the production of progeny virus. Indeed, MCMV replication in macrophages was highly reduced when m38.5 or m41.1 was deleted from the viral genome. Moreover, the Δm38.5 mutant exhibited a growth defect in endothelial cells and dendritic cells [55,56]. However, both deletion mutants showed only a moderate replication defect when propagated on fibroblasts [52,55,56]. Surprisingly, the Δm38.5 mutant grew to similar titers as the wildtype virus in visceral organs such as spleen, liver, and lungs of infected mice. By contrast, viral titers were clearly reduced in the salivary glands and, to a lesser extent, in splenic leukocytes [55]. As leukocytes of the monocyte/macrophage lineage are important for dissemination of MCMV to different organs [58], it was suggested that the reduced salivary gland titers might be a direct consequence of the reduced replication of the mutant virus in leukocytes [55]. In HCMV the influence of vMIA on virus replication appears to depend on the virus strain. Deletion of UL37x1 from the Towne strain had almost no impact on virus replication in fibroblasts [43], whereas deletion of UL37x1 from the AD169 (variant ATCC) strain resulted in a virus that induced apoptosis and showed a strong replication deficit in fibroblasts [59,60]. This phenotype was reversed by treatment of infected cells with the caspase-inhibitor zVAD-fmk, suggesting that caspase-dependent apoptosis was primarily responsible for the observed replication deficit [59]. The reason for the different ΔUL37x1 phenotypes remains unknown. Possibly, the inactivation of the cell death inhibitor vICA in AD169*var*ATCC (see section below) might have an impact on the replication capability. Another explanation could be that the AD169*var*ATCC strain produces a stronger pro-apoptotic stimulus in infected cells than the Towne strain [43]. 

Intriguingly, HCMV blocks apoptosis at the level of mitochondria not only by the protein vMIA but also by a viral RNA transcript [61]. The unspliced polyadenylated β2.7 RNA is abundantly transcribed in infected cells but does not code for a protein [62,63,64]. The expression of the β2.7 RNA conferred protection of cells from apoptosis induced by the pesticide rotenone or by ischemia/reperfusion, both of which compromise the function of the mitochondrial respiratory complex I [61,65]. This complex is localized at the inner mitochondrial membrane and is an enzyme of the respiratory transport chain, the main source of ATP production. Inhibition of complex I leads to a reduction of cellular ATP levels, increase of mitochondrial ROS production and ultimately to mitochondria-mediated apoptosis [66,67]. Immunoprecipitation experiments revealed that the β2.7 RNA interacts with native complex I and specifically with its essential subunit GRIM19 (gene associated with retinoid/interferon-induced mortality 19) [61]. During viral infection the β2.7 RNA conferred protection against rotenone-induced reduction of ATP production and was required for sustained ATP production at late times postinfection (in the absence of rotenone) [61]. The β2.7 RNA was also able to reduce ROS production [65]. Together, these data suggested that the interaction of the β2.7 RNA with complex I leads to a stabilization of the complex, resulting in the maintenance of ATP production and sustained cell viability. 

## 3. Inhibition of Death Receptor-Induced Apoptosis

Extrinsic death signals are mediated via death receptors, such as the TNFR1 (tumor necrosis factor receptor 1), TRAILR (TNF-related apoptosis inducing ligand receptor) or Fas. Stimulation of these receptors leads to their oligomerization and to the assembly of a DISC at the cytosolic part of the receptors. The DISC is composed of several proteins including FADD (FAS-associated protein with a death domain), RIP1 and c-FLIPs (cellular FLICE-inhibitory protein) [68,69,70,71,72]. This protein complex subsequently recruits procaspase-8, which is then activated by autoproteolytic cleavage [73,74]. In type I cells (e.g., lymphocytes) caspase-8 directly cleaves effector caspase-3 leading to execution of apoptosis. In type II cells (e.g., hepatocytes) the execution phase follows the mitochondria-dependent pathway [5,75,76]. In that case, DISC-activated caspase-8 cleaves the BH3-only protein Bid. Truncated Bid (tBid) then translocates to mitochondria leading to MOMP and subsequent execution of apoptosis [77,78] (Figure 1).

HCMV blocks the induction of extrinsic apoptosis by its protein UL36. UL36’s anti-apoptotic function was first revealed by screening an HCMV expression library for proteins capable of blocking Fas-induced apoptosis in HeLa cells [79]. UL36 also prevented apoptosis triggered by TNFα and TRAIL. Cells infected with UL36-mutated or UL36-deficient virus were less resistant to Fas- and TNFR-induced apoptosis than wildtype virus-infected cells at early time points postinfection (*i.e.*, 24 hpi) [79,80]. Co-immunoprecipitation analysis revealed that UL36 interacts with procaspase-8 and inhibits its proteolytic processing and thus its activation [79]. Consequently, UL36 was named viral inhibitor of caspase-8-induced apoptosis (vICA). vICA interacts with the pro-domain of caspase‑8 [79], which also mediates interaction with FADD [81]. However, an interaction of vICA with FADD was not observed [79], suggesting a model in which vICA inhibits the recruitment of procaspase-8 to FADD, thereby preventing the formation of a functional DISC. 

Most or all mammalian betaherpesviruses encode sequence homologs of HCMV vICA, implying that the function of vICA is important and conserved [82]. Indeed, for MCMV and rhesus macaque CMV the anti-apoptotic function of their vICAs (M36 and Rh36, respectively) has already been demonstrated, and MCMV vICA was also shown to interact with procaspase-8 [49,83]. For replication in cultured fibroblasts, HCMV vICA is not required [84]. Interestingly, the two extensively fibroblast-passaged HCMV laboratory stains AD169*var*ATCC and Towne*var*RIT carry mutated vICAs lacking anti-apoptotic activity [79], suggesting that inactivation of vICA might even be beneficial for viral replication in cultured fibroblasts or virus release from infected fibroblasts. Similarly, vICA of the rhesus CMV strain 68-1 carries an inactivating mutation [49], which does not seem to affect pathogenicity of this virus strain in fetal macaques [85]. In contrast to the lack of a replication phenotype in fibroblasts, the anti-apoptotic function of HCMV vICA is required for efficient replication in cells of the monocyte-macrophage lineage since vICA deficient viruses induced apoptosis and exhibited impaired virus growth in these cells, which was rescued by treatment with the caspase-inhibitor zVAD-fmk [80]. Similarly, a previous study has shown that an MCMV mutant lacking vICA grew to normal titers in fibroblasts while titers in macrophages were reduced. In these cells the vICA-deficient virus induced caspase-8 activation and apoptosis [83]. Since macrophages are important for MCMV dissemination in mice [58], one would expect a vICA-deficient virus to be attenuated *in vivo*. Indeed, a vICA-deficient MCMV reached reduced titers in liver, lungs, and salivary glands, and induced apoptosis in infected tissue macrophages and hepatocytes [86]. Insertion of a dominant negative version of FADD into the genome of the ΔM36 virus compensated for the loss of vICA *in vitro* and largely rescued virus replication *in vivo* [86]. These results confirmed that inhibition of FADD-dependent extrinsic apoptosis is the main function of vICA and revealed the importance of this function for viral fitness

## 4. Inhibition of Programmed Necrosis

**Figure 2:**
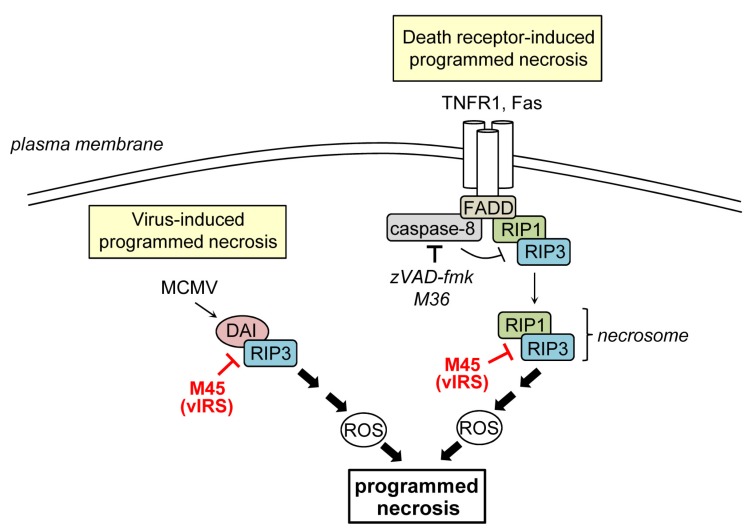
Inhibition of programmed necrosis by murine CMV. Death receptor-induced programmed necrosis is mediated by RIP1 and RIP3 and can be activated when caspase-8 activity is blocked. M45 binds to RIP1 and RIP3, inhibits their complex formation, and the induction of death receptor-induced programmed necrosis. M45 also suppresses virus‑induced programmed necrosis, which is mediated by DAI and RIP3, both of which are targeted by M45.

Programmed necrosis is a regulated form of cell death which cannot be blocked by caspase-inhibitors and is, therefore, a caspase-independent form of PCD. Programmed necrosis can be triggered following stimulation of various receptors including the death receptors TNFR1 and Fas [12,87] or following stimulation with Poly[I:C], LPS, or CpG DNA, which are activators of the pattern recognition receptors TLR3, -4, -and -9, respectively [88,89,90,91]. Moreover, it can be initiated by several viruses and has recently been recognized as an important antiviral host defense mechanism [92,93,94,95,96]. The intracellular signaling pathways regulating programmed necrosis have only recently begun to be unraveled. Most of the current knowledge has been derived from studies on TNFR1-induced programmed necrosis. Usually, TNFR1 ligation induces apoptosis via the formation of a protein complex that includes TRADD (TNFR1-associated death domain protein), FADD, RIP1 and caspase-8 [8]. However, when caspase-8 activation is inhibited, death receptor stimulation can trigger programmed necrosis [10,97]. Necrosis induction is dependent on the activity of RIP1 and RIP3, two related kinases that contain a RIP homotypic interaction motif (RHIM) [96,98,99,100]. It seems that caspase-inhibition triggers programmed necrosis because caspase-8 acts as a negative regulator of necrosis that cleaves RIP1 and RIP3 thereby preventing necrosis [9,101,102,103]. During necrosis induction, RIP1 recruits RIP3 to build a complex, the so-called necrosome [14,100] (Figure 2). The interaction of both proteins is mediated by RIP homotypic interaction motifs (RHIMs) and is stabilized by phosphorylation [8,96,104]. Following necrosome formation RIP3 recruits downstream substrates such as MLKL (mixed lineage kinase domain-like protein) and the mitochondrial phosphatase PGAM5 (phosphoglycerate mutase family member 5). PGAM5 in turn binds and activates dynamin-related protein 1 (Drp1), a GTPase that controls mitochondrial fragmentation, a process that occurs early during necrosis [105,106]. How these events finally cause necrosis is currently unknown. Important players during necrosis execution are calcium and ROS, which directly or indirectly cause damage of cellular constituents finally leading to the disruption of organelles and the plasma membrane [6,107].

The first cell death inhibitor identified in MCMV is the protein encoded by ORF M45 [108]. The initial study showed that M45 inhibits infection-induced cell death: mutant viruses carrying a transposon insertion or a frame-shift mutation in M45 rapidly induced PCD of cultured endothelial cells and macrophages thereby precluding viral spread [108]. On the molecular level, M45 was shown to interact with RIP1 and block caspase-independent TNFR1- and Fas-induced PCD, *i.e.*, programmed necrosis [109]. This activity is likely of major importance for the virus given the fact that MCMV encodes a caspase-8 inhibitor (see above), which should render infected cells sensitive to programmed necrosis. M45 also inhibited RIP1-dependent activation of p38 MAP kinase and the transcription factor NF-κB [109]. Consequently the protein was named viral inhibitor of RIP-mediated signaling (vIRS) [109] or, by others, viral inhibitor of RIP1 activation (vIRA) [110]. Subsequent publications demonstrated that M45 carries a RHIM near its N-terminus, which is required for inhibition of RIP1‑RIP3 complex formation and TNFR1-induced programmed necrosis [95,110].

In terms of M45-mutant virus-induced PCD it was demonstrated that cell death induction could not be rescued by the caspase-inhibitor zVAD-fmk, did not require TNFα, and dying cells showed typical necrotic features [95]. These results indicated that cell death was of necrotic nature and was not induced by the TNFR1 but by the virus itself. M45-mutant virus triggered programmed necrosis not only in SVEC endothelial cells and macrophages but also in 3T3-SA fibroblasts, whereas other fibroblasts, such as NIH 3T3 cells, were more resistant [95,109]. An important step towards understanding the differences in sensitivity to virus-induced programmed necrosis has been made by studies demonstrating that elevated expression levels of RIP3 and DAI (DNA-dependent activator of interferon regulatory factors (IRFs)) confer sensitivity to MCMV-induced programmed necrosis. Indeed, SVEC and 3T3-SA cells contain high levels of both proteins, whereas NIH 3T3 fibroblasts contain little amounts of RIP3 and apparently lack DAI [95,98,111]. Furthermore, RIP1 was found to be dispensable for MCMV-induced necrosis, showing that death receptor-induced and virus-induced programmed necrosis do not rely on an identical set of cellular proteins [95]. DAI is a RHIM containing cytoplasmic DNA sensor originally described as an activator of IRFs and NF-κB [112,113]. During M45-mutant virus-induced necrosis DAI interacts with RIP3, providing evidence that a RIP3‑DAI complex mediates necrotic signals [111]. M45 binds to RIP3 and DAI and compromises their interaction [110,111,113]. When the RHIM of M45 is mutated in MCMV (M45*mut*RHIM), the virus loses its ability to inhibit RIP3-DAI complex formation and virus-induced necrosis [95]. Thus, the M45 RHIM is essential to inhibit both, programmed necrosis induced by death receptor ligation or by virus infection.

A recent study has shown that M45 also interacts with the NF-κB essential modulator (NEMO), the regulatory subunit of the IKK complex that is responsible for activation of NF-κB. M45 inhibits NF‑κB activation by targeting NEMO to autophagosomes for subsequent degradation in lysosomes. By depleting this essential signaling molecule, the virus blocks the production of proinflammatory cytokines such as TNFα and IL-6 [114]. Interestingly, NEMO has recently also been implicated in mediating necrotic cell death signals upon TNFα stimulation. In that case, NEMO appears to act downstream of the RIP1-RIP3 complex, independently of NF-κB [115]. Thus, in addition to blocking necrotic signals at the level of RIP1 and RIP3, the M45-induced degradation of NEMO might also contribute to the inhibition of TNFR1-induced programmed necrosis.

The importance of M45 for viral fitness and pathogenesis was demonstrated by *in vivo* studies. MCMV mutants expressing truncated M45 proteins were non-pathogenic even in immunocompromised SCID mice, which are usually highly susceptible to MCMV and die within 4 weeks after infection. Furthermore, no virus replication could be detected in target organs such as spleen, liver, lungs or salivary glands on day 24 postinfection [116]. Necrosis inhibition *in vivo* also requires a functional M45 RHIM. This became clear when growth properties of the M45*mut*RHIM virus were analyzed. Compared to wildtype virus this mutant yielded lower viral titers in liver, spleen, and salivary glands, and was not pathogenic. Importantly, this phenotype was reversed in RIP3 knockout mice and attenuated in DAI-deficient mice, confirming that inhibition of RIP3/DAI-mediated cell death is a crucial function of M45 during infection [95,111]. 

M45 shows homology to the large subunit of the ribonucleotide reductase (R1), an enzyme that is required for dNTP synthesis. The homology domain lies within the C-terminal part of M45 and is devoid of catalytic R1 function [116,117,118]. It seems that the C-terminus has gained a new function, since only a small portion of it can be deleted without losing the ability to inhibit TNFR1-, TLR (Toll‑like receptor)- and IL-1R-induced NF-κB activation [109,114]. The C-terminal part also plays a role for cell death inhibition as it was observed that MCMVs with C-terminally truncated M45 induced cell death [108] and the N-terminal part alone provided only partial protection against cell death [110]. Thus, both parts of M45 are required for full protection against cell death induction. 

HCMV possesses a sequence homolog to M45, which is encoded by ORF UL45. The UL45 protein also contains a catalytically inactive R1 homology domain but does not have a RHIM in its N-terminal part. Unlike M45, UL45 is dispensable for virus replication in endothelial cells [119]. A minor replication defect of a UL45-deficient HCMV was detected on fibroblasts after low-m.o.i. infection as compared to infection with the parental wildtype virus [120]. The same study showed that mutant virus-infected cells were slightly more sensitive to Fas-induced cell death. However, when UL45 was expressed alone by retroviral transduction it did not protect fibroblasts against Fas-induced cell death [120]. Therefore it seems that UL45 does not share M45’s cell death-inhibiting functions. Further studies are needed to define the function of UL45 and its role during HCMV infection.

## 5. Inhibition of Stress-Induced Cell Death

The intrinsic apoptotic pathway can be induced by different stress conditions like ER stress or DNA damage. ER stress is triggered, for instance, by an overload of unfolded proteins within the organelle or by perturbation in Ca^2+^ homeostasis. This results in the activation of the unfolded protein response (UPR), which aims to resolve ER stress and to keep the cell vital. However, when stress conditions persist for an extended time and cannot be resolved, the UPR induces apoptosis [121]. This pathway can be mediated, among others, by the ER stress sensor IRE1 and the downstream c-Jun N-terminal kinase (JNK) [122]. Upon IRE1 activation JNK is recruited and activated by phosphorylation [123]. Activated JNK in turn inhibits anti-apoptotic Bcl-2 and activates pro-apoptotic BH3-only proteins thereby inducing the mitochondrial apoptosis pathway [123,124,125] (Figure 1). 

HCMV infection induces the UPR but, at the same time, the virus modulates its outcome [126]. The HCMV UL38 protein plays an important role in this process. Viruses lacking UL38 massively induced caspase-dependent apoptosis in fibroblasts at late times postinfection [127]. Isolated overexpression of UL38 protected cells from apoptosis induced by the ER stress inducers thapsigargin and tunicamycin or by infection with E1B-19K-deficient adenovirus, which induces the intrinsic apoptosis pathway [127,128]. In terms of the underlying molecular mechanisms it was shown that UL38 inhibits persistent JNK phosphorylation, *i.e.*, activation. Chemical inhibition of JNK activation enhanced the viability of cells infected with UL38-deficient virus which provided strong evidence that JNK is involved in mediating cell death induced by this mutant virus [128]. Moreover, UL38 induced an accumulation of ATF4 (activating transcriptional factor 4) [128], a transcription factor whose translation is induced following activation of the ER-stress sensor PERK (protein kinase R-like ER kinase) [129]. Upon overexpression ATF4 was capable of enhancing viability of cells infected with UL38-deficient virus, indicating that the UL38-induced accumulation of ATF4 contributes to the ability of UL38 to suppress cell death [128]. UL38 has also been described to interact with TSC2 (tuberous sclerosis protein 2) thereby maintaining signaling of mTORC1 (mammalian target of rapamycin complex 1), a protein complex that promotes protein synthesis and is usually inactivated by several metabolic stress conditions [130]. Analysis of UL38 truncation mutants recently revealed that the anti-apoptotic function of UL38 can be separated from its role in mTORC1 activation [131]. 

It has recently been described that MCMV regulates the UPR similar to HCMV [132]. However, in case of MCMV, the mechanisms of regulation are largely unknown. MCMV encodes a sequence homolog to UL38 but it has not been reported so far whether or not the MCMV homolog, M38, shares any functional similarities with UL38.

## 6. Summary and Conclusions

The induction of PCD in infected cells is a crucial host cell mechanism to curtail infection within the organism. PCD can be induced by numerous stimuli, including cytokines, microbial structures stimulating pattern recognition receptors, DNA damage, or ER stress. It has been described that CMV infection leads to the production of different cytokines [133,134]. Moreover, several TLRs seem to be activated during CMV infection [135,136,137,138] and DNA damage as well as ER stress are induced by CMV [126,132,139]. Thus, CMV provides many triggers for the induction of PCD. Therefore it is not surprising that the virus encodes a series of cell death inhibitors. Apoptosis is blocked at different levels by CMV (Figure 1): vICA inhibits caspase-8 activation thus intervening at an early step of the extrinsic apoptosis pathway. vMIA and vIBO inhibit apoptosis at the mitochondrial checkpoint by targeting the two executioner proteins Bax and Bak, respectively. Blocking apoptosis at this level provides protection not only against intrinsic apoptotic stimuli but, in many cells types (type II cells), also against extrinsically induced apoptosis. Programmed necrosis, an alternative cell death pathway that is activated when caspase-8 is inactivated, is also blocked by MCMV. Inhibition is accomplished by the protein vIRS, which targets RIP1, RIP3, and DAI for this purpose (Figure 2). It is remarkable that CMV attacks converging points within the cell death pathways and that many of the viral cell death inhibitors have additional functions. This strategy allows the virus to face numerous antiviral pathways with a limited set of viral genes. Reducing the genetic burden by this means is a strategy typically followed by small viruses, which have only limited coding capacity. Apparently, the large herpesviruses also follow this strategy to some extent, suggesting that limiting the viral genetic content might be beneficial for viruses irrespective of their genome size.

Interestingly, none of the CMV cell death inhibitors show homology to cellular genes involved in cell death regulation. This is in contrast to other viruses, including γ-herpesviruses, which encode viral Bcl-2s and viral FLIPs that show homology to cellular anti-apoptotic proteins, indicating that they were captured from the host genome [140]. Why the CMVs have evolved their own inhibitors remains enigmatic. 

Examining the function of viral cell death inhibitors expands our knowledge on antiviral strategies and cellular cell death pathways. Good examples are the studies on vIRS, which showed that the induction of programmed necrosis is a physiological mechanism used by the host to restrict virus infection. The complete abrogation of MCMV replication in certain cell types and pathogenesis in the mouse due to the induction of necrosis provides strong evidence that this cell death mode is at least as important as apoptosis in terms of antiviral defense. This notion is supported by a report, which showed that vaccinia virus infection is also controlled by a programmed necrosis-induced inflammatory response [96]. Importantly, these studies not only revealed that programmed necrosis is a crucial antiviral mechanism but also defined which cellular proteins (primarily RIP3 and DAI) regulate this process in the context of MCMV infection. 

It is likely that further cell death inhibitors will be discovered in the genomes of CMVs. There are also viral proteins whose mechanisms of cell death inhibition are yet unknown, as is the case for the MCMV protein m41, a Golgi-localized protein [141]. Clarifying the function of these genes and identifying further cell death inhibitors will expand our knowledge on host-virus interaction. Finally, this might also contribute to the generation of novel antiviral drugs: Suppressing the anti-apoptotic and anti-necrotic functions of viral proteins by specifically targeted compounds would allow infected cells to undergo PCD and thus be depleted from the organism.

## References

[1] Elmore S. (2007). Apoptosis: A review of programmed cell death. Toxicol. Pathol..

[2] Hacker G. (2000). The morphology of apoptosis. Cell Tissue Res..

[3] Galluzzi L., Vitale I., Abrams J.M., Alnemri E.S., Baehrecke E.H., Blagosklonny M.V., Dawson T.M., Dawson V.L., El-Deiry W.S., Fulda S. (2012). Molecular definitions of cell death subroutines: Recommendations of the Nomenclature Committee on Cell Death 2012. Cell Death Differ..

[4] Kumar S. (2007). Caspase function in programmed cell death. Cell Death Differ..

[5] Scaffidi C., Fulda S., Srinivasan A., Friesen C., Li F., Tomaselli K.J., Debatin K.M., Krammer P.H., Peter M.E. (1998). Two CD95 (APO-1/Fas) signaling pathways. EMBO J..

[6] Festjens N., Vanden Berghe T., Vandenabeele P. (2006). Necrosis, a well-orchestrated form of cell demise: Signalling cascades, important mediators and concomitant immune response. Biochim. Biophys. Acta.

[7] Zong W.X., Thompson C.B. (2006). Necrotic death as a cell fate. Genes Dev..

[8] Vandenabeele P., Galluzzi L., Vanden Berghe T., Kroemer G. (2010). Molecular mechanisms of necroptosis: An ordered cellular explosion. Nat. Rev. Mol. Cell Biol..

[9] Chan F.K., Shisler J., Bixby J.G., Felices M., Zheng L., Appel M., Orenstein J., Moss B., Lenardo M.J. (2003). A role for tumor necrosis factor receptor-2 and receptor-interacting protein in programmed necrosis and antiviral responses. J. Biol. Chem..

[10] Vercammen D., Beyaert R., Denecker G., Goossens V., Van Loo G., Declercq W., Grooten J., Fiers W., Vandenabeele P. (1998). Inhibition of caspases increases the sensitivity of L929 cells to necrosis mediated by tumor necrosis factor. J. Exp. Med..

[11] Han J., Zhong C.Q., Zhang D.W. (2011). Programmed necrosis: backup to and competitor with apoptosis in the immune system. Nat. Immunol..

[12] Laster S.M., Wood J.G., Gooding L.R. (1988). Tumor necrosis factor can induce both apoptic and necrotic forms of cell lysis. J. Immunol..

[13] Vanlangenakker N., Vanden Berghe T., Vandenabeele P. (2012). Many stimuli pull the necrotic trigger, an overview. Cell Death Differ..

[14] Declercq W., Vanden Berghe T., Vandenabeele P. (2009). RIP kinases at the crossroads of cell death and survival. Cell.

[15] Kroemer G., Levine B. (2008). Autophagic cell death: the story of a misnomer. Nat. Rev. Mol. Cell Biol..

[16] Cannon M.J., Schmid D.S., Hyde T.B. (2010). Review of cytomegalovirus seroprevalence and demographic characteristics associated with infection. Rev. Med. Virol..

[17] Steininger C. (2007). Clinical relevance of cytomegalovirus infection in patients with disorders of the immune system. Clin. Microbiol. Infect..

[18] Hudson J.B. (1979). The murine cytomegalovirus as a model for the study of viral pathogenesis and persistent infections. Arch. Virol..

[19] Krmpotic A., Bubic I., Polic B., Lucin P., Jonjic S. (2003). Pathogenesis of murine cytomegalovirus infection. Microbes Infect..

[20] Voigt S., Ettinger J., Streblow D.N., Reddehase M.J. (2013). The rat model of cytomegalovirus infection and vascular disease. Cytomegaloviruses: From Molecular Pathogenesis to Intevention.

[21] Brune W. (2011). Inhibition of programmed cell death by cytomegaloviruses. Virus Res..

[22] Kroemer G., Galluzzi L., Brenner C. (2007). Mitochondrial membrane permeabilization in cell death. Physiol. Rev..

[23] Riedl S.J., Salvesen G.S. (2007). The apoptosome: signalling platform of cell death. Nat. Rev. Mol. Cell Biol..

[24] Ow Y.P., Green D.R., Hao Z., Mak T.W. (2008). Cytochrome c: Functions beyond respiration. Nat. Rev. Mol. Cell Biol..

[25] Youle R.J., Strasser A. (2008). The BCL-2 protein family: Opposing activities that mediate cell death. Nat. Rev. Mol. Cell Biol..

[26] Bouillet P., Strasser A. (2002). BH3-only proteins—Evolutionarily conserved proapoptotic Bcl-2 family members essential for initiating programmed cell death. J. Cell Sci..

[27] Uren R.T., Dewson G., Chen L., Coyne S.C., Huang D.C., Adams J.M., Kluck R.M. (2007). Mitochondrial permeabilization relies on BH3 ligands engaging multiple prosurvival Bcl-2 relatives, not Bak. J. Cell Biol..

[28] Willis S.N., Fletcher J.I., Kaufmann T., van Delft M.F., Chen L., Czabotar P.E., Ierino H., Lee E.F., Fairlie W.D., Bouillet P. (2007). Apoptosis initiated when BH3 ligands engage multiple Bcl-2 homologs, not Bax or Bak. Science.

[29] Gavathiotis E., Suzuki M., Davis M.L., Pitter K., Bird G.H., Katz S.G., Tu H.C., Kim H., Cheng E.H., Tjandra N. (2008). BAX activation is initiated at a novel interaction site. Nature.

[30] Kuwana T., Bouchier-Hayes L., Chipuk J.E., Bonzon C., Sullivan B.A., Green D.R., Newmeyer D.D. (2005). BH3 domains of BH3-only proteins differentially regulate Bax-mediated mitochondrial membrane permeabilization both directly and indirectly. Mol. Cell.

[31] Letai A., Bassik M.C., Walensky L.D., Sorcinelli M.D., Weiler S., Korsmeyer S.J. (2002). Distinct BH3 domains either sensitize or activate mitochondrial apoptosis, serving as prototype cancer therapeutics. Cancer Cell.

[32] Nechushtan A., Smith C.L., Hsu Y.T., Youle R.J. (1999). Conformation of the Bax C-terminus regulates subcellular location and cell death. EMBO J..

[33] Antonsson B., Montessuit S., Sanchez B., Martinou J.C. (2001). Bax is present as a high molecular weight oligomer/complex in the mitochondrial membrane of apoptotic cells. J. Biol. Chem..

[34] Wei M.C., Lindsten T., Mootha V.K., Weiler S., Gross A., Ashiya M., Thompson C.B., Korsmeyer S.J. (2000). tBID, a membrane-targeted death ligand, oligomerizes BAK to release cytochrome c. Genes Dev..

[35] Dewson G., Kratina T., Sim H.W., Puthalakath H., Adams J.M., Colman P.M., Kluck R.M. (2008). To trigger apoptosis, Bak exposes its BH3 domain and homodimerizes via BH3:groove interactions. Mol. Cell.

[36] Dewson G., Kratina T., Czabotar P., Day C.L., Adams J.M., Kluck R.M. (2009). Bak activation for apoptosis involves oligomerization of dimers via their alpha6 helices. Mol. Cell.

[37] Westphal D., Dewson G., Czabotar P.E., Kluck R.M. (2011). Molecular biology of Bax and Bak activation and action. Biochim. Biophys. Acta.

[38] Belzacq A.S., El Hamel C., Vieira H.L., Cohen I., Haouzi D., Metivier D., Marchetti P., Brenner C., Kroemer G. (2001). Adenine nucleotide translocator mediates the mitochondrial membrane permeabilization induced by lonidamine, arsenite and CD437. Oncogene.

[39] Jan G., Belzacq A.S., Haouzi D., Rouault A., Metivier D., Kroemer G., Brenner C. (2002). Propionibacteria induce apoptosis of colorectal carcinoma cells via short-chain fatty acids acting on mitochondria. Cell Death Differ..

[40] Arnoult D., Bartle L.M., Skaletskaya A., Poncet D., Zamzami N., Park P.U., Sharpe J., Youle R.J., Goldmacher V.S. (2004). Cytomegalovirus cell death suppressor vMIA blocks Bax- but not Bak-mediated apoptosis by binding and sequestering Bax at mitochondria. Proc. Natl. Acad. Sci. U. S. A..

[41] Goldmacher V.S., Bartle L.M., Skaletskaya A., Dionne C.A., Kedersha N.L., Vater C.A., Han J.W., Lutz R.J., Watanabe S., Cahir McFarland E.D. (1999). A cytomegalovirus-encoded mitochondria-localized inhibitor of apoptosis structurally unrelated to Bcl-2. Proc. Natl. Acad. Sci. U. S. A..

[42] Boya P., Cohen I., Zamzami N., Vieira H.L., Kroemer G. (2002). Endoplasmic reticulum stress-induced cell death requires mitochondrial membrane permeabilization. Cell Death Differ..

[43] McCormick A.L., Meiering C.D., Smith G.B., Mocarski E.S. (2005). Mitochondrial cell death suppressors carried by human and murine cytomegalovirus confer resistance to proteasome inhibitor-induced apoptosis. J. Virol..

[44] Pauleau A.L., Larochette N., Giordanetto F., Scholz S.R., Poncet D., Zamzami N., Goldmacher V.S., Kroemer G. (2007). Structure-function analysis of the interaction between Bax and the cytomegalovirus-encoded protein vMIA. Oncogene.

[45] Poncet D., Larochette N., Pauleau A.L., Boya P., Jalil A.A., Cartron P.F., Vallette F., Schnebelen C., Bartle L.M., Skaletskaya A. (2004). An anti-apoptotic viral protein that recruits Bax to mitochondria. J. Biol. Chem..

[46] Hayajneh W.A., Colberg-Poley A.M., Skaletskaya A., Bartle L.M., Lesperance M.M., Contopoulos-Ioannidis D.G., Kedersha N.L., Goldmacher V.S. (2001). The sequence and antiapoptotic functional domains of the human cytomegalovirus UL37 exon 1 immediate early protein are conserved in multiple primary strains. Virology.

[47] Smith G.B., Mocarski E.S. (2005). Contribution of GADD45 family members to cell death suppression by cellular Bcl-xL and cytomegalovirus vMIA. J. Virol..

[48] McCormick A.L., Roback L., Mocarski E.S. (2008). HtrA2/Omi terminates cytomegalovirus infection and is controlled by the viral mitochondrial inhibitor of apoptosis (vMIA). PLoS Pathog..

[49] McCormick A.L., Skaletskaya A., Barry P.A., Mocarski E.S., Goldmacher V.S. (2003). Differential function and expression of the viral inhibitor of caspase 8-induced apoptosis (vICA) and the viral mitochondria-localized inhibitor of apoptosis (vMIA) cell death suppressors conserved in primate and rodent cytomegaloviruses. Virology.

[50] Norris K.L., Youle R.J. (2008). Cytomegalovirus proteins vMIA and m38.5 link mitochondrial morphogenesis to Bcl-2 family proteins. J. Virol..

[51] Andoniou C.E., Andrews D.M., Manzur M., Ricciardi-Castagnoli P., Degli-Esposti M.A. (2004). A novel checkpoint in the Bcl-2-regulated apoptotic pathway revealed by murine cytomegalovirus infection of dendritic cells. J. Cell Biol..

[52] Jurak I., Schumacher U., Simic H., Voigt S., Brune W. (2008). Murine cytomegalovirus m38.5 protein inhibits Bax-mediated cell death. J. Virol..

[53] Degenhardt K., Sundararajan R., Lindsten T., Thompson C., White E. (2002). Bax and Bak independently promote cytochrome C release from mitochondria. J. Biol. Chem..

[54] Wei M.C., Zong W.X., Cheng E.H., Lindsten T., Panoutsakopoulou V., Ross A.J., Roth K.A., MacGregor G.R., Thompson C.B., Korsmeyer S.J. (2001). Proapoptotic BAX and BAK: A requisite gateway to mitochondrial dysfunction and death. Science.

[55] Manzur M., Fleming P., Huang D.C., Degli-Esposti M.A., Andoniou C.E. (2009). Virally mediated inhibition of Bax in leukocytes promotes dissemination of murine cytomegalovirus. Cell Death Differ..

[56] Cam M., Handke W., Picard-Maureau M., Brune W. (2010). Cytomegaloviruses inhibit Bak- and Bax-mediated apoptosis with two separate viral proteins. Cell Death Differ..

[57] Karbowski M., Norris K.L., Cleland M.M., Jeong S.Y., Youle R.J. (2006). Role of Bax and Bak in mitochondrial morphogenesis. Nature.

[58] Hanson L.K., Slater J.S., Karabekian Z., Virgin H.W.t., Biron C.A., Ruzek M.C., van Rooijen N., Ciavarra R.P., Stenberg R.M., Campbell A.E. (1999). Replication of murine cytomegalovirus in differentiated macrophages as a determinant of viral pathogenesis. J. Virol..

[59] Reboredo M., Greaves R.F., Hahn G. (2004). Human cytomegalovirus proteins encoded by UL37 exon 1 protect infected fibroblasts against virus-induced apoptosis and are required for efficient virus replication. J. Gen. Virol..

[60] Yu D., Silva M.C., Shenk T. (2003). Functional map of human cytomegalovirus AD169 defined by global mutational analysis. Proc. Natl. Acad. Sci. U. S. A..

[61] Reeves M.B., Davies A.A., McSharry B.P., Wilkinson G.W., Sinclair J.H. (2007). Complex I binding by a virally encoded RNA regulates mitochondria-induced cell death. Science.

[62] McDonough S.H., Staprans S.I., Spector D.H. (1985). Analysis of the major transcripts encoded by the long repeat of human cytomegalovirus strain AD169. J. Virol..

[63] Greenaway P.J., Wilkinson G.W. (1987). Nucleotide sequence of the most abundantly transcribed early gene of human cytomegalovirus strain AD169. Virus Res..

[64] McSharry B.P., Tomasec P., Neale M.L., Wilkinson G.W. (2003). The most abundantly transcribed human cytomegalovirus gene (beta 2.7) is non-essential for growth *in vitro*. J. Gen. Virol..

[65] Zhao J., Sinclair J., Houghton J., Bolton E., Bradley A., Lever A. (2010). Cytomegalovirus beta2.7 RNA transcript protects endothelial cells against apoptosis during ischemia/reperfusion injury. J. Heart Lung Transplant..

[66] Li N., Ragheb K., Lawler G., Sturgis J., Rajwa B., Melendez J.A., Robinson J.P. (2003). Mitochondrial complex I inhibitor rotenone induces apoptosis through enhancing mitochondrial reactive oxygen species production. J. Biol. Chem..

[67] Hoglinger G.U., Carrard G., Michel P.P., Medja F., Lombes A., Ruberg M., Friguet B., Hirsch E.C. (2003). Dysfunction of mitochondrial complex I and the proteasome: Interactions between two biochemical deficits in a cellular model of Parkinson's disease. J. Neurochem..

[68] Kischkel F.C., Hellbardt S., Behrmann I., Germer M., Pawlita M., Krammer P.H., Peter M.E. (1995). Cytotoxicity-dependent APO-1 (Fas/CD95)-associated proteins form a death-inducing signaling complex (DISC) with the receptor. EMBO J..

[69] Sprick M.R., Weigand M.A., Rieser E., Rauch C.T., Juo P., Blenis J., Krammer P.H., Walczak H. (2000). FADD/MORT1 and caspase-8 are recruited to TRAIL receptors 1 and 2 and are essential for apoptosis mediated by TRAIL receptor 2. Immunity.

[70] Scaffidi C., Schmitz I., Krammer P.H., Peter M.E. (1999). The role of c-FLIP in modulation of CD95-induced apoptosis. J. Biol. Chem..

[71] Schutze S., Tchikov V., Schneider-Brachert W. (2008). Regulation of TNFR1 and CD95 signalling by receptor compartmentalization. Nat. Rev. Mol. Cell Biol..

[72] Morgan M.J., Kim Y.S., Liu Z.G. (2009). Membrane-bound Fas ligand requires RIP1 for efficient activation of caspase-8 within the death-inducing signaling complex. J. Immunol..

[73] Muzio M., Chinnaiyan A.M., Kischkel F.C., O'Rourke K., Shevchenko A., Ni J., Scaffidi C., Bretz J.D., Zhang M., Gentz R. (1996). FLICE, a novel FADD-homologous ICE/CED-3-like protease, is recruited to the CD95 (Fas/APO-1) death—Inducing signaling complex. Cell.

[74] Medema J.P., Scaffidi C., Kischkel F.C., Shevchenko A., Mann M., Krammer P.H., Peter M.E. (1997). FLICE is activated by association with the CD95 death-inducing signaling complex (DISC). EMBO J..

[75] Yin X.M., Wang K., Gross A., Zhao Y., Zinkel S., Klocke B., Roth K.A., Korsmeyer S.J. (1999). Bid-deficient mice are resistant to Fas-induced hepatocellular apoptosis. Nature.

[76] Barnhart B.C., Alappat E.C., Peter M.E. (2003). The CD95 type I/type II model. Semin. Immunol..

[77] Li H., Zhu H., Xu C.J., Yuan J. (1998). Cleavage of BID by caspase 8 mediates the mitochondrial damage in the Fas pathway of apoptosis. Cell.

[78] Luo X., Budihardjo I., Zou H., Slaughter C., Wang X. (1998). Bid, a Bcl2 interacting protein, mediates cytochrome c release from mitochondria in response to activation of cell surface death receptor. Cell.

[79] Skaletskaya A., Bartle L.M., Chittenden T., McCormick A.L., Mocarski E.S., Goldmacher V.S. (2001). A cytomegalovirus-encoded inhibitor of apoptosis that suppresses caspase-8 activation. Proc. Natl. Acad. Sci. U. S. A..

[80] McCormick A.L., Roback L., Livingston-Rosanoff D., St Clair C. (2010). The human cytomegalovirus UL36 gene controls caspase-dependent and -independent cell death programs activated by infection of monocytes differentiating to macrophages. J. Virol..

[81] Carrington P.E., Sandu C., Wei Y., Hill J.M., Morisawa G., Huang T., Gavathiotis E., Werner M.H. (2006). The structure of FADD and its mode of interaction with procaspase-8. Mol. Cell.

[82] McCormick A.L., Skaletskaya A., Barry P.A., Mocarski E.S., Goldmacher V.S. (2003). Differential function and expression of the viral inhibitor of caspase 8-induced apoptosis (vICA) and the viral mitochondria-localized inhibitor of apoptosis (vMIA) cell death suppressors conserved in primate and rodent cytomegaloviruses. Virology.

[83] Menard C., Wagner M., Ruzsics Z., Holak K., Brune W., Campbell A.E., Koszinowski U.H. (2003). Role of murine cytomegalovirus US22 gene family members in replication in macrophages. J. Virol..

[84] Patterson C.E., Shenk T. (1999). Human cytomegalovirus UL36 protein is dispensable for viral replication in cultured cells. J. Virol..

[85] Chang W.L., Tarantal A.F., Zhou S.S., Borowsky A.D., Barry P.A. (2002). A recombinant rhesus cytomegalovirus expressing enhanced green fluorescent protein retains the wild-type phenotype and pathogenicity in fetal macaques. J. Virol..

[86] Cicin-Sain L., Ruzsics Z., Podlech J., Bubic I., Menard C., Jonjic S., Reddehase M.J., Koszinowski U.H. (2008). Dominant-negative FADD rescues the *in vivo* fitness of a cytomegalovirus lacking an antiapoptotic viral gene. J. Virol..

[87] Holler N., Zaru R., Micheau O., Thome M., Attinger A., Valitutti S., Bodmer J.L., Schneider P., Seed B., Tschopp J. (2000). Fas triggers an alternative, caspase-8-independent cell death pathway using the kinase RIP as effector molecule. Nat. Immunol..

[88] Kalai M., Van Loo G., Vanden Berghe T., Meeus A., Burm W., Saelens X., Vandenabeele P. (2002). Tipping the balance between necrosis and apoptosis in human and murine cells treated with interferon and dsRNA. Cell Death Differ..

[89] Ma Y., Temkin V., Liu H., Pope R.M. (2005). NF-kappaB protects macrophages from lipopolysaccharide-induced cell death: the role of caspase 8 and receptor-interacting protein. J. Biol. Chem..

[90] Lalanne A.I., Moraga I., Hao Y., Pereira J.P., Alves N.L., Huntington N.D., Freitas A.A., Cumano A., Vieira P. (2010). CpG inhibits pro-B cell expansion through a cathepsin B-dependent mechanism. J. Immunol..

[91] He S., Liang Y., Shao F., Wang X. (2011). Toll-like receptors activate programmed necrosis in macrophages through a receptor-interacting kinase-3-mediated pathway. Proc. Natl. Acad. Sci. U. S. A..

[92] Lenardo M.J., Angleman S.B., Bounkeua V., Dimas J., Duvall M.G., Graubard M.B., Hornung F., Selkirk M.C., Speirs C.K., Trageser C. (2002). Cytopathic killing of peripheral blood CD4(+) T lymphocytes by human immunodeficiency virus type 1 appears necrotic rather than apoptotic and does not require env. J. Virol..

[93] Petit F., Arnoult D., Lelievre J.D., Moutouh-de Parseval L., Hance A.J., Schneider P., Corbeil J., Ameisen J.C., Estaquier J. (2002). Productive HIV-1 infection of primary CD4+ T cells induces mitochondrial membrane permeabilization leading to a caspase-independent cell death. J. Biol. Chem..

[94] Peri P., Nuutila K., Vuorinen T., Saukko P., Hukkanen V. (2011). Cathepsins are involved in virus-induced cell death in ICP4 and Us3 deletion mutant herpes simplex virus type 1-infected monocytic cells. J. Gen. Virol..

[95] Upton J.W., Kaiser W.J., Mocarski E.S. (2010). Virus inhibition of RIP3-dependent necrosis. Cell Host Microbe.

[96] Cho Y.S., Challa S., Moquin D., Genga R., Ray T.D., Guildford M., Chan F.K. (2009). Phosphorylation-driven assembly of the RIP1-RIP3 complex regulates programmed necrosis and virus-induced inflammation. Cell.

[97] Vercammen D., Brouckaert G., Denecker G., Van de Craen M., Declercq W., Fiers W., Vandenabeele P. (1998). Dual signaling of the Fas receptor: Initiation of both apoptotic and necrotic cell death pathways. J. Exp. Med..

[98] Zhang D.W., Shao J., Lin J., Zhang N., Lu B.J., Lin S.C., Dong M.Q., Han J. (2009). RIP3, an energy metabolism regulator that switches TNF-induced cell death from apoptosis to necrosis. Science.

[99] Vandenabeele P., Declercq W., Van Herreweghe F., Vanden Berghe T. (2010). The role of the kinases RIP1 and RIP3 in TNF-induced necrosis. Sci. Signal..

[100] He S., Wang L., Miao L., Wang T., Du F., Zhao L., Wang X. (2009). Receptor interacting protein kinase-3 determines cellular necrotic response to TNF-alpha. Cell.

[101] Feng S., Yang Y., Mei Y., Ma L., Zhu D.E., Hoti N., Castanares M., Wu M. (2007). Cleavage of RIP3 inactivates its caspase-independent apoptosis pathway by removal of kinase domain. Cell Signal.

[102] Oberst A., Dillon C.P., Weinlich R., McCormick L.L., Fitzgerald P., Pop C., Hakem R., Salvesen G.S., Green D.R. (2011). Catalytic activity of the caspase-8-FLIP(L) complex inhibits RIPK3-dependent necrosis. Nature.

[103] Gunther C., Martini E., Wittkopf N., Amann K., Weigmann B., Neumann H., Waldner M.J., Hedrick S.M., Tenzer S., Neurath M.F. (2011). Caspase-8 regulates TNF-alpha-induced epithelial necroptosis and terminal ileitis. Nature.

[104] Sun X., Yin J., Starovasnik M.A., Fairbrother W.J., Dixit V.M. (2002). Identification of a novel homotypic interaction motif required for the phosphorylation of receptor-interacting protein (RIP) by RIP3. J. Biol. Chem..

[105] Sun L., Wang H., Wang Z., He S., Chen S., Liao D., Wang L., Yan J., Liu W., Lei X. (2012). Mixed lineage kinase domain-like protein mediates necrosis signaling downstream of RIP3 kinase. Cell.

[106] Wang Z., Jiang H., Chen S., Du F., Wang X. (2012). The mitochondrial phosphatase PGAM5 functions at the convergence point of multiple necrotic death pathways. Cell.

[107] Vanlangenakker N., Vanden Berghe T., Krysko D.V., Festjens N., Vandenabeele P. (2008). Molecular mechanisms and pathophysiology of necrotic cell death. Curr. Mol. Med..

[108] Brune W., Menard C., Heesemann J., Koszinowski U.H. (2001). A ribonucleotide reductase homolog of cytomegalovirus and endothelial cell tropism. Science.

[109] Mack C., Sickmann A., Lembo D., Brune W. (2008). Inhibition of proinflammatory and innate immune signaling pathways by a cytomegalovirus RIP1-interacting protein. Proc. Natl. Acad. Sci. U. S. A..

[110] Upton J.W., Kaiser W.J., Mocarski E.S. (2008). Cytomegalovirus M45 cell death suppression requires receptor-interacting protein (RIP) homotypic interaction motif (RHIM)-dependent interaction with RIP1. J. Biol. Chem..

[111] Upton J.W., Kaiser W.J., Mocarski E.S. (2012). DAI/ZBP1/DLM-1 complexes with RIP3 to mediate virus-induced programmed necrosis that is targeted by murine cytomegalovirus vIRA. Cell Host Microbe.

[112] Takaoka A., Wang Z., Choi M.K., Yanai H., Negishi H., Ban T., Lu Y., Miyagishi M., Kodama T., Honda K. (2007). DAI (DLM-1/ZBP1) is a cytosolic DNA sensor and an activator of innate immune response. Nature.

[113] Rebsamen M., Heinz L.X., Meylan E., Michallet M.C., Schroder K., Hofmann K., Vazquez J., Benedict C.A., Tschopp J. (2009). DAI/ZBP1 recruits RIP1 and RIP3 through RIP homotypic interaction motifs to activate NF-kappaB. EMBO Rep..

[114] Fliss P.M., Jowers T.P., Brinkmann M.M., Holstermann B., Mack C., Dickinson P., Hohenberg H., Ghazal P., Brune W. (2012). Viral mediated redirection of NEMO/IKKgamma to autophagosomes curtails the inflammatory cascade. PLoS Pathog..

[115] Irrinki K.M., Mallilankaraman K., Thapa R.J., Chandramoorthy H.C., Smith F.J., Jog N.R., Gandhirajan R.K., Kelsen S.G., Houser S.R., May M.J. (2011). Requirement of FADD, NEMO, and BAX/BAK for aberrant mitochondrial function in tumor necrosis factor alpha-induced necrosis. Mol. Cell Biol..

[116] Lembo D., Donalisio M., Hofer A., Cornaglia M., Brune W., Koszinowski U., Thelander L., Landolfo S. (2004). The ribonucleotide reductase R1 homolog of murine cytomegalovirus is not a functional enzyme subunit but is required for pathogenesis. J. Virol..

[117] Rawlinson W.D., Farrell H.E., Barrell B.G. (1996). Analysis of the complete DNA sequence of murine cytomegalovirus. J. Virol..

[118] Lembo D., Brune W. (2009). Tinkering with a viral ribonucleotide reductase. Trends Biochem. Sci..

[119] Hahn G., Khan H., Baldanti F., Koszinowski U.H., Revello M.G., Gerna G. (2002). The human cytomegalovirus ribonucleotide reductase homolog UL45 is dispensable for growth in endothelial cells, as determined by a BAC-cloned clinical isolate of human cytomegalovirus with preserved wild-type characteristics. J. Virol..

[120] Patrone M., Percivalle E., Secchi M., Fiorina L., Pedrali-Noy G., Zoppe M., Baldanti F., Hahn G., Koszinowski U.H., Milanesi G. (2003). The human cytomegalovirus UL45 gene product is a late, virion-associated protein and influences virus growth at low multiplicities of infection. J. Gen. Virol..

[121] Chakrabarti A., Chen A.W., Varner J.D. (2011). A review of the mammalian unfolded protein response. Biotechnol. Bioeng..

[122] Szegezdi E., Logue S.E., Gorman A.M., Samali A. (2006). Mediators of endoplasmic reticulum stress-induced apoptosis. EMBO Rep..

[123] Urano F., Wang X., Bertolotti A., Zhang Y., Chung P., Harding H.P., Ron D. (2000). Coupling of stress in the ER to activation of JNK protein kinases by transmembrane protein kinase IRE1. Science.

[124] Lei K., Davis R.J. (2003). JNK phosphorylation of Bim-related members of the Bcl2 family induces Bax-dependent apoptosis. Proc. Natl. Acad. Sci. U. S. A..

[125] Putcha G.V., Le S., Frank S., Besirli C.G., Clark K., Chu B., Alix S., Youle R.J., LaMarche A., Maroney A.C. (2003). JNK-mediated BIM phosphorylation potentiates BAX-dependent apoptosis. Neuron.

[126] Isler J.A., Skalet A.H., Alwine J.C. (2005). Human cytomegalovirus infection activates and regulates the unfolded protein response. J. Virol..

[127] Terhune S., Torigoi E., Moorman N., Silva M., Qian Z., Shenk T., Yu D. (2007). Human cytomegalovirus UL38 protein blocks apoptosis. J. Virol..

[128] Xuan B., Qian Z., Torigoi E., Yu D. (2009). Human cytomegalovirus protein pUL38 induces ATF4 expression, inhibits persistent JNK phosphorylation, and suppresses endoplasmic reticulum stress-induced cell death. J. Virol..

[129] Harding H.P., Novoa I., Zhang Y., Zeng H., Wek R., Schapira M., Ron D. (2000). Regulated translation initiation controls stress-induced gene expression in mammalian cells. Mol. Cell.

[130] Moorman N.J., Cristea I.M., Terhune S.S., Rout M.P., Chait B.T., Shenk T. (2008). Human cytomegalovirus protein UL38 inhibits host cell stress responses by antagonizing the tuberous sclerosis protein complex. Cell Host Microbe.

[131] Qian Z., Xuan B., Gualberto N., Yu D. (2011). The human cytomegalovirus protein pUL38 suppresses endoplasmic reticulum stress-mediated cell death independently of its ability to induce mTORC1 activation. J. Virol..

[132] Qian Z., Xuan B., Chapa T.J., Gualberto N., Yu D. (2012). Murine cytomegalovirus targets transcription factor ATF4 to exploit the unfolded-protein response. J. Virol..

[133] Ruzek M.C., Miller A.H., Opal S.M., Pearce B.D., Biron C.A. (1997). Characterization of early cytokine responses and an interleukin (IL)-6-dependent pathway of endogenous glucocorticoid induction during murine cytomegalovirus infection. J. Exp. Med..

[134] Orange J.S., Biron C.A. (1996). Characterization of early IL-12, IFN-alphabeta, and TNF effects on antiviral state and NK cell responses during murine cytomegalovirus infection. J. Immunol..

[135] Compton T., Kurt-Jones E.A., Boehme K.W., Belko J., Latz E., Golenbock D.T., Finberg R.W. (2003). Human cytomegalovirus activates inflammatory cytokine responses via CD14 and Toll-like receptor 2. J. Virol..

[136] Kijpittayarit S., Eid A.J., Brown R.A., Paya C.V., Razonable R.R. (2007). Relationship between Toll-like receptor 2 polymorphism and cytomegalovirus disease after liver transplantation. Clin. Infect. Dis..

[137] Tabeta K., Georgel P., Janssen E., Du X., Hoebe K., Crozat K., Mudd S., Shamel L., Sovath S., Goode J. (2004). Toll-like receptors 9 and 3 as essential components of innate immune defense against mouse cytomegalovirus infection. Proc. Natl. Acad. Sci. U. S. A..

[138] Delale T., Paquin A., Asselin-Paturel C., Dalod M., Brizard G., Bates E.E., Kastner P., Chan S., Akira S., Vicari A. (2005). MyD88-dependent and -independent murine cytomegalovirus sensing for IFN-alpha release and initiation of immune responses *in vivo*. J. Immunol..

[139] Gaspar M., Shenk T. (2006). Human cytomegalovirus inhibits a DNA damage response by mislocalizing checkpoint proteins. Proc. Natl. Acad. Sci. U. S. A..

[140] Galluzzi L., Brenner C., Morselli E., Touat Z., Kroemer G. (2008). Viral control of mitochondrial apoptosis. PLoS Pathog..

[141] Brune W., Nevels M., Shenk T. (2003). Murine cytomegalovirus m41 open reading frame encodes a Golgi-localized antiapoptotic protein. J. Virol..

